# Prevalence of Premorbid Metabolic Syndrome in Spanish Adult Workers Using IDF and ATPIII Diagnostic Criteria: Relationship with Cardiovascular Risk Factors

**DOI:** 10.1371/journal.pone.0089281

**Published:** 2014-02-20

**Authors:** Pedro Tauler, Miquel Bennasar-Veny, Jose M. Morales-Asencio, Angel A. Lopez-Gonzalez, Teofila Vicente-Herrero, Joan De Pedro-Gomez, Vanessa Royo, Jordi Pericas-Beltran, Antoni Aguilo

**Affiliations:** 1 Fundamental Biology and Health Sciences Department, Universitat Illes Balears, Palma, Spain; 2 Research Group on Evidence, Lifestyles & Health, Universitat Illes Balears, Palma, Spain; 3 Prevention of Occupational Risks in Health Services, Balearic Islands Health Service, Palma, Spain; 4 Prevention of Occupational Risks, Correos, Valencia, Spain; 5 Gastroenterology Services, Manacor Hospital, Manacor, Spain; Virgen Macarena University Hospital, School of Medicine, University of Seville, Spain

## Abstract

**Background:**

Metabolic Syndrome (MetS) is a complex disorder defined as a cluster of interconnected risk factors such as hypertension, dyslipidemia, obesity and high blood glucose levels. Premorbid metabolic syndrome (PMetS) is defined by excluding patients with previously diagnosed cardiovascular disease or diabetes mellitus from those suffering MetS. We aimed to determine the prevalence of PMetS in a working population, and to analyse the relationship between the diagnostic criteria of the International Diabetes Federation (IDF) and of the National Cholesterol Education Program Adult Treatment Panel III (ATPIII). The relationship between the presence of PMetS and cardiovascular risk factors was also analysed.

**Research Methodology/Findings:**

A cross-sectional study was conducted in 24,529 male and 18,736 female Spanish (white western European) adult workers (20–65 years) randomly selected during their work health periodic examinations. Anthropometrics, blood pressure and serum parameters were measured. The presence of MetS and PMetS was ascertained using ATPIII and IDF criteria. Cardiovascular risk was determined using the Framingham-REGICOR equation. The results showed MetS had an adjusted global prevalence of 12.39% using ATPIII criteria and 16.46% using IDF criteria. The prevalence of PMetS was slightly lower (11.21% using ATPIII criteria and 14.72% using IDF criteria). Prevalence in males was always higher than in females. Participants with PMetS displayed higher values of BMI, waist circumference, blood pressure, glucose and triglycerides, and lower HDL-cholesterol levels. Logistic regression models reported lower PMetS risk for females, non-obese subjects, non-smokers and younger participants. Cardiovascular risk determined with Framingham-REGICOR was higher in participants with PMetS.

**Conclusions:**

PMetS could be a reliable tool for the early identification of apparently healthy individuals who have a significant risk for developing cardiovascular events and type 2 diabetes.

## Introduction

Cardiovascular diseases (CVD), diabetes mellitus and obesity are some of the main public health challenges in the 21^st^ century [1]–[3]. Metabolic Syndrome (MetS) is a complex disorder with a high socioeconomic cost [4] which is defined as a cluster of interconnected risk factors such as hypertension, dyslipidemia, obesity and high blood glucose levels, leading to increased risk of developing cardiovascular diseases and type 2 diabetes [5]–[8]. Nowadays MetS is becoming a worldwide epidemic because of the rise in obesity prevalence and a sedentary lifestyle. In fact, the prevalence of MetS in the adult population is relatively high [4], [9]. This prevalence is influenced by several factors such as age, gender, lifestyles, socioeconomic variables and ethnicity [10]–[13].

Since the concept of MetS was introduced in 1988 by the World Health Organization (WHO) [14], several modifications have been included in the parameters used to determine the presence of MetS [4], [15]–[17]. Nowadays, following the general consensus of WHO, the two most widely used definitions are those of the National Cholesterol Education Program Adult Treatment Panel III (ATPIII) and the International Diabetes Federation (IDF) [18]. The ATPIII set of criteria to determine the presence of MetS includes waist circumference (WC), blood lipids, blood pressure and fasting glucose [19]. Meanwhile, the IDF definition includes the occurrence of central (abdominal) obesity together with decreased HDL-cholesterol, increased triglycerides, increased blood pressure and hyperglycaemia. Using these criteria, a prevalence of 13–30% in developing countries and approximately 30–35% in developed countries is usually found [20]–[24]. In Spain, several epidemiological studies have determined the prevalence of MetS [2], [25]–[27], some of which used the new WHO criteria [2], [27]. Furthermore, a few studies have focused on the working population [28]–[30]. However, to the best of our knowledge, comparisons between the prevalence obtained using IDF and ATPIII criteria are not found in the literature.

It is striking that WHO suggested excluding individuals who already have diabetes mellitus or CVD from the previous definition of MetS, because MetS cannot be used for primary prevention in these individuals [31]. This new condition, derived from the WHO suggestion, is termed premorbid metabolic syndrome (PMetS), and its prevalence and impact are as yet unknown.

The risk factors related to MetS include health-related behaviours such as smoking, alcohol consumption and lack of exercise [32], [33]. ATPIII places major emphasis on lifestyle changes as the main aim in the therapeutic approach for clinical management of people at risk for CVD [34]. In fact, although the prevalence of cardiovascular risk factors (CVRF) has been widely reported from the results of the Framingham study, CVRF prevalence in relation to MetS has not been clearly described. Modification of lifestyle-related behaviours toward healthier habits, e.g. improving dietary habits or increasing exercise practice, could be an essential, non-medical, strategy for MetS treatment [9]. In fact, several studies have reported beneficial effects of interventions focused on promoting healthier habits in the workplace [35]–[37].

The aims of this study were to determine the prevalence of PMetS in a working population, and to analyse the relationship between the diagnostic criteria of the IDF and ATPIII, and between the presence of PMetS and cardiovascular risk factors. The accomplishment of these aims will enable us to know the distribution of CVD risk factors in the working population and, thus, to establish adequate strategies for health promotion in the workplace.

## Materials and Methods

### Design and analytic sample

The present study is based on cross-sectional data from 43,265 working white western European adults (20–65 years) from the Balearic Islands (Spain) belonging to different productive sectors (public administration, health department and post offices). Participants in the study were randomly selected during their periodic health examination in the workplace. Every day each worker was assigned a number and half of the examined workers were randomly selected using a random number table. 54,236 workers were invited to participate in the study. However, 10,971 (20.2%) refused to participate, being the final number of participants 43,265 (56.7% males and 43.3% females). This sample represents the 9.45% of the total working active population from the Balearic Islands in 2011 [38]. The accuracy obtained with this sample was 0.53% for an alpha value of 0.05, a reference active population in 2011 of 457,750 subjects [38] and considering a MetS prevalence of 10.2% [28]. Taking into account the crude prevalence found in the present study the accuracy was 0.48%.

The study protocol was in accordance with the Declaration of Helsinki and was approved by the by the Institutional Review Board of the Mallorca Health Management Ethical Review Committee of GESMA. Participants were informed of the purpose of this study before they provided written consent to participate. After acceptance, a self-reported complete medical history, including family and personal history, was recorded. Occupational data were also recorded [39]. This study was conducted between January 2008 and December 2010. The following inclusion criteria were considered: age between 20 and 65 years (working age population) and being gainfully employed. Subjects who did not meet any of the inclusion criteria were excluded from the study. The data included in the present manuscript is freely available upon request to the corresponding author.

### Data collection and definition of variables

The methodology used was similar to the one previously reported [40]. All anthropometric measurements were made according to the recommendations of the International Standards for Anthropometric Assessment (ISAK) [41]. Furthermore, all measurements were performed by experienced technicians to minimize coefficients of variation and each measurement was made three times and the average value was calculated.

Weight and height were determined according to recommended techniques mentioned above. Body weight was measured to the nearest 0.1 kg using an electronic scale (Seca 700 scale, Seca gmbh, Hamburg). Height was measured to the nearest 0.5 cm using a stadiometer (Seca 220 (CM) Telescopic Height Rod for Column Scales, Seca gmbh, Hamburg). BMI was calculated as weight (kg) divided by height (m) squared (kg/m^2^). Criteria used to define overweight were the ones of the World Health Organization (WHO), the US Preventive Services Task Force and the International Obesity Task Force which define obesity as a BMI ≥30 kg/m^2^ [42].

Abdominal waist and hip circumferences were measured using a flexible steel tape (Lufkin Executive Thinline W 606). The plane of the tape was perpendicular to the long axis of the body and parallel to the floor. Waist circumference was measured half-way between the lower costal border and the iliac crest. The measurement was made at the end of a normal expiration while the subject stood upright, with feet together and arms hanging freely at the sides. Hip circumference was measured over non-restrictive underwear, or light-weight shorts, at the level of the maximum extension of the buttocks posteriorly in a horizontal plane, without compressing the skin.

Venous blood samples were taken from the antecubital vein with suitable vacutainers without anticoagulant to obtain serum. Blood samples were taken following a 12 h overnight fast. Participants were seated at rest for at least 15 minutes before blood samples were taken. Serum was obtained after centrifugation (15 min, 1,000×g, 4°C) of blood samples. Serum was stored at −20°C and analyses were performed within 3 days. Concentrations of glucose, cholesterol and triglycerides were measured in serum by standard procedures used in clinical biochemistry laboratory by using an autoanalyser (SYNCHRON CX®9 PRO, Beckman Coulter, Brea, CA, USA).

Blood pressure was determined after a resting period of 10 minutes in the supine position using an automatic and calibrated sphygmomanometer (OMRON M3, OMRON Healthcare Europe, Spain). As indicated for the anthropometrical measures, blood pressure was measured three times with a one-minute gap between each measurement and an average value was calculated.

The presence of MetS was ascertained by using the criterion suggested by ATPIII and IDF. Characteristics included in the ATPIII definition are:

- Abdominal obesity (given as waist circumference, males>102 cm and females >88 cm)
- Triglycerides ≥150 mg/dL
- HDL-cholesterol <40 mg/dL in males and <50 mg/dL in females
- Blood pressure ≥130/85 mm Hg
- Fasting glucose ≥100 mg/dL

When three of the five listed characteristics were present a diagnosis of metabolic syndrome was made [43].Characteristics included in the IDF definition are:

- Central obesity (defined as waist circumference, males ≥94 cm and females ≥80 cm; when BMI is >30 kg/m^2^, central obesity can be assumed and waist circumference does not need to be measured)
- Triglycerides ≥150 mg/dL or specific treatment for this lipid abnormality
- HDL-cholesterol <40 mg/dL in males and <50 mg/dL in females
- Blood pressure ≥130/85 mm Hg or treatment for previously diagnosed hypertension
- Fasting plasma glucose >100 mg/dL.

When central obesity plus two of the four previous criteria were met, a diagnosis of MetS was made [18].

The PMetS group was obtained from the participants with MetS by excluding those with a previous diagnostic of CVD or type 2 diabetes [31]. The information regarding the previous diagnostic of CVD or type 2 diabetes, as well as for the pharmacological treatments, was self-reported.

The REGICOR-Framingham risk equation, which supposes an adaptation to the cardiovascular risk factors prevalence and cardiovascular events characteristics in the Spanish population [44], was used to determine the cardiovascular risk.

### Statistical analyses

Descriptive statistics via exploratory analysis were performed using central trend and scatter measures for continuous variables and analysis of proportions for categorical variables. We carried out analysis of the type of distribution and normality test for each variable using the Kolmogorov-Smirnov, together with Q-Q normal probability plots. For bivariate analysis Student’s t-test for means in normal distribution variables (using the Levene test for variance equality) and non-parametric tests such as the U Mann-Whitney test (independent samples) and Wilcoxon test (paired data) for variables showing non-normal distribution were used. For categorical variables the chi squared test and Fisher’s exact test whenever necessary for each contingency table were used. We also computed correlation and regression measures when necessary for continuous variables. Additionally, ANOVA tests with the post-hoc Bonferroni contrast method were carried out.

Prevalence was analysed with crude and adjusted values for gender and age. For this purpose we used the Balearic population figures from the Spanish National Statistics Institute. All the results were described with their corresponding 95% confidence intervals. Correlations were calculated among waist circumference and the other components of the metabolic syndrome. Framingham-REGICOR risk scores were calculated both with ATP-III and IDF criteria to determine the association of different criteria with cardiovascular risk, through adjusted OR by sex and gender. The agreement between IDF and ATPIII criteria was analysed by means of Kappa coefficient determination.

Multivariate analysis was performed by logistic regression to evaluate the contribution to PMetS of the following risk factors: age, gender, BMI and smoking status. Two regression models were designed, a first one for the ATP III criteria and a second one for the IDF criteria. Goodness-of-fit tests for the model (–2 log-likelihood, goodness-of-fit statistic, Cox and Snell R^2^, and Nagelkerke R^2^) were calculated to assess the global adjustment of the model. Exponentiation was used for the β-coefficients in the regression models to estimate the OR, and the standard error of the β-coefficients was used to calculate the 95% CIs of the RR estimates by published methods.

Statistical analysis was carried out using IBM SPSS Statistics 20.0 software (SPSS/IBM, Chicago, IL, USA). Significance was accepted at p<0.05.

## Results

### Characteristics of the participants in the study

Table 1 shows the general characteristics of the participants in the study. Male participants had higher values of blood pressure, total cholesterol, triglycerides and glucose and lower levels of HDL-cholesterol (p<0.001) than females. Overweight was present in 35.5% of the participants, and obesity in 16.2%. From the sample, 5.1% of participants were on medication for hypertension and 2.5% for dyslipidemia. Among participants in the study, 35.5% were smokers.

**Table 1:** General characteristics of the participants categorized by gender.

	Total (n = 43,265)	Male (n = 24,529; 57.0%)	Female (n = 18,736; 43%)	
	Mean (95% CI) or n (%)	Mean (95% CI) or n (%)	Mean (95% CI) or n (%)	p
Smoker	15,360 (35.5)	9,104 (59.3)	6,256 (40.7)	<0.001
Non smoker	20,583 (47.6)	10,624 (51.6)	9,959 (48.4)	<0.001
Ex-smoker	7,322 (16.9)	4,801 (65.6)	2,521(34.4)	<0.001
Age (years)	39.28 (39.18 to 39.37)	39.63 (39.50 to 39.76)	38.82 (38.67 to 38.96)	<0.001
BMI (kg/m^2^)	25.81 (25.76 to 25.85)	26.73 (26.68 to 26.78)	24.6 (24.54 to 24.67)	<0.001
SBP (mm Hg)	120.49 (120.34 to 120.64)	125.4 (125.21 to 125.59)	114.07 (113.86 to 114.28)	<0.001
DBP (mm Hg)	73.31 (73.21 to 73.41)	75.72 (75.58 to 75.85)	70.16 (70.02 to 70.31)	<0.001
Cholesterol (g/L)	195.22(191.87 to 192.57)	194.25 (193.77 to 194.73)	189.57 (189.06 to 190.09)	<0.001
HDL-cholesterol (g/L)	51.80 (51.72 to 51.88)	49.9 (49.82 to 49.99)	54.28 (54.15 to 54.42)	<0.001
Glucose (g/L)	86.11 (85.98 to 86.24)	88.28 (88.09 to 88.47)	83.27 (83.11 to 83.44)	<0.001
Triglycerides (g/L)	104.87 (104.17 to 105.56)	121.38 (120.29 to 122.48)	83.24 (82.63 to 83.85)	<0.001
WC (cm)	82.22 (82.12 to 82.33)	88.09 (87.97 to 88.21)	74.55 (74.42 to 74.68)	<0.001

BMI: Body mass index; SBP: Systolic bold pressure; DBP: Diastolic blood pressure; HDL: High density lipoproteins; WC: Waist circumference.

### Prevalence of MetS and PMetS

The crude and the adjusted prevalence of MetS were determined, using both the ATPIII and the IDF criteria, for the whole population and for the participants categorized by gender (Table S1). The adjusted global prevalence of MetS with ATPIII criteria was 12.39% (21.39% in males and 6.94% in females). Using the IDF criteria, the adjusted global prevalence was 16.46%, higher in males (28.42%) than in females (10.07%).

**Figure 1:**
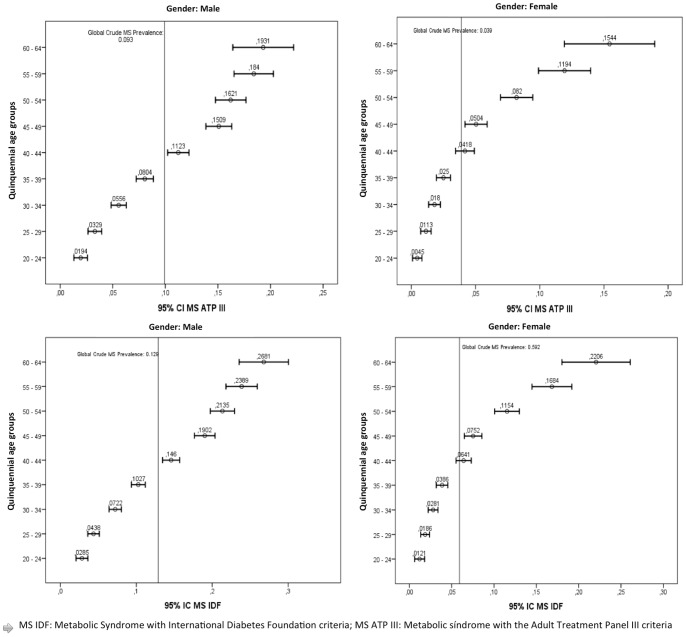
Prevalence of premorbid metabolic syndrome following the ATP III and the IDF criteria by ages (p<0.001).

The detailed crude and adjusted prevalence of PMetS by gender and age is shown in Table 2. For both genders and for all the age groups prevalence of PMetS was higher using the IDF criteria than using the ATPIII ones. Furthermore, when all the parameters included in the PMetS diagnostic criteria were analysed, all of them were significantly higher in males than in females, except for HDL and BMI, which were slightly higher in females (Table 3). In addition, the analysis by quinquennial age groups showed that PMetS prevalence increased with age (Figure 1).

**Table 2:** Crude and adjusted* prevalence of PMetS in Balearic working population with ATPIII and IDF criteria.

	CRUDE						ADJUSTED								
ATP III	Global n (%) (n = 43,265)	95% CI	Male n (%) (n = 24,529)	95% CI	Female n (%) (n = 18,736)	95% CI	Global (%) (n = 43,265)	95% CI		Male (%) (n = 24,529)		95% CI		Female (%) (n = 18,736)	95% CI
**20–24 (n = 3,077)**	40 (1.3)	1.1 to 1.5	34 (1.94)	1.61 to 2.27	6 (0.45)	0.27 to 0.64	1.65%	1.39 to 1.91		2.49%		2.07 to 2.91		0.57%	0.34 to 0.8
**25–29 (n = 5,428)**	123 (2.27)	2.06 to 2.47	94 (3.29)	2.96 to 3.63	29 (1.13)	0.92 to 1.33	3.81%	3.47 to 4.15		5.13%		4.62 to 5.65		2.08%	1.7 to 2.46
**30–34 (n = 7,074)**	274 (3.87)	3.64 to 4.1	217 (5.56)	5.19 to 5.92	57 (1.8)	1.56 to 2.03	6.82%	6.42 to 7.22		9.31%		8.71 to 9.91		3.36%	2.92 to 3.79
**35–39 (n = 7,257)**	413 (5.69)	5.42 to 5.96	336 (8.04)	7.62 to 8.46	77 (2.5)	2.22 to 2.78	10.04%	9.57 to 10.5		13.71%		13.02 to 14.41		4.55%	4.04 to 5.06
**40–44 (n = 6,631)**	542 (8.17)	7.84 to 8.51	422 (11.23)	10.71 to 11.74	120 (4.18)	3.8 to 4.55	14.63%	14.05 to 15.21		19.32%		18.48 to 20.17		7.79%	7.11 to 8.47
**45–49 (n = 5,728)**	613 (10.7)	10.29 to 11.11	487 (15.09)	14.46 to 15.72	126 (5.04)	4.6 to 5.48	18.31%	17.64 to 18.98		25.14%		24.16 to 26.13		8.87%	8.12 to 9.63
**50–54 (n = 4,321)**	554 (12.82)	12.31 to 13.33	404 (16.21)	15.47 to 16.95	150 (8.2)	7.56 to 8.84	19.14%	18.41 to 19.88		24.58%		23.52 to 25.64		11.99%	11.07 to 12.91
**55–59 (n = 2,621)**	419 (15.99)	15.27 to 16.7	302 (18.4)	17.45 to 19.36	117 (11.94)	10.9 to 12.97	17.06%	16.3 to 17.82		21.78%		20.67 to 22.89		10.95%	10 to 11.91
**60–64 (n = 1,128)**	202 (17.91)	16.77 to 19.05	139 (19.31)	17.83 to 20.78	63 (15.44)	13.65 to 17.23	9.23%	8.61 to 9.85		11.36%		10.45 to 12.26		6.55%	5.76 to 7.35
**GLOBAL**	**3,180 (7.35)**	**7.22 to 7.48**	**2,435 (9.93)**	**9.74 to 10.12**	**745 (3.98)**	**3.83 to 4.12**	**11.21%**	**11.02 to 11.39**		**19.44%**		**19.13 to 19.75**		**6.17%**	**5.95 to 6.39**
	**CRUDE**						**ADJUSTED**								
**IDF**	**Global n (%) (n = 43,265)**	**95% CI**	**Male n (%) (n = 24,529)**	**95% CI**	**Female n (%) (n = 18,736)**	**95% CI**	**Global (%) (n = 43,265)**		**95% CI**		**Male (%) (n = 24,529)**		**95% CI**	**Female (%) (n = 18,736)**	**95% CI**
**20–24 (n = 3,077)**	66 (2.13)	1.87 to 2.39	50 (2.85)	2.46 to 3.25	16 (1.21)	0.91 to 1.51	2.66%		2.34 to 2.99		3.66%		3.15 to 4.17	1.52%	1.14 to 1.9
**25–29 (n = 5,428)**	173 (3.16)	2.93 to 3.4	125 (4.38)	4 to 4.76	48 (1.86)	1.6 to 2.13	5.24%		4.85 to 5.62		6.82%		6.24 to 7.41	3.44%	2.95 to 3.93
**30–34 (n = 7,074)**	371 (5.2)	4.94 to 5.47	282 (7.22)	6.81 to 7.64	89 (2.81)	2.51 to 3.1	9.02%		8.57 to 9.47		12.10%		11.42 to 12.77	5.24%	4.7 to 5.78
**35–39 (n = 7,257)**	548 (7.47)	7.16 to 7.78	429 (10.27)	9.8 to 10.74	119 (3.86)	3.52 to 4.21	13.01%		12.49 to 13.53		17.51%		16.74 to 18.28	7.03%	6.41 to 7.65
**40–44 (n = 6,631)**	733 (10.9)	10.52 to 11.28	549 (14.6)	14.03 to 15.18	184 (6.41)	5.95 to 6.86	19.33%		18.69 to 19.97		25.14%		24.21 to 26.07	11.95%	11.12 to 12.77
**45–49 (n = 5,728)**	802 (13.59)	13.15 to 14.04	614 (19.02)	18.33 to 19.71	188 (7.52)	6.99 to 8.05	23.40%		22.68 to 24.12		31.70%		30.64 to 32.76	13.24%	12.34 to 14.14
**50–54 (n = 4,321)**	743 (16.35)	15.81 to 16.9	532 (21.35)	20.53 to 22.17	211 (11.54)	10.79 to 12.28	25.08%		24.29 to 25.88		32.37%		31.21 to 33.52	16.87%	15.81 to 17.93
**55–59 (n = 2,621)**	557 (19.65)	18.91 to 20.4	392 (23.89)	22.84 to 24.94	165 (16.84)	15.64 to 18.03	22.15%		21.32 to 22.98		28.27%		27.06 to 29.48	15.44%	14.34 to 16.55
**60–64 (n = 1,128)**	283 (22.44)	21.27 to 23.62	193 (26.81)	25.15 to 28.46	90 (22.06)	20.01 to 24.11	12.63%		11.93 to 13.33		15.77%		14.73 to 16.81	9.36%	8.42 to 10.3
**GLOBAL**	**4,276 (9.65)**	**9.51 to 9.8**	**3,166 (12.91)**	**12.69 to 13.12**	**1,110 (5.92)**	**5.75 to 6.1**	**14.72%**		**14.51 to 14.93**		**26.15%**		**25.8 to 26.49**	**9.19%**	**8.93 to 9.45**

*Adjusted by Balearic population. PMetS: premorbid metabolic syndrome.

**Table 3:** Distribution of risk factors by the presence/absence of PMetS.

Premorbid metabolic syndrome (ATP III Criteria)					
		No n = 22,094	Yes n = 2,435		
		Median (95%CI)	Median (95%CI)	p	
Male	BMI (kg/m^2^)	26.29 (26.24 to 26.34)	30.71 (30.53 to 30.89)	<0.001	
	SBP (mm Hg)	124.03 (123.84 to 124.22)	137.82 (137.2 to 138.44)	<0.001	
	DBP (mm Hg)	74.81 (74.68 to 74.95)	83.94 (83.51 to 84.36)	<0.001	
	Cholesterol (g/L)	190.76 (190.28 to 191.24)	225.89 (224.26 to 227.53)	<0.001	
	HDL-cholesterol (g/L)	50.75 (50.67 to 50.83)	42.22 (41.91 to 42.52)	<0.001	
	Glucose (g/L)	87.01 (86.83 to 87.19)	99.76 (98.84 to 100.69)	<0.001	
	Triglycerides (g/L)	105.53 (104.74 to 106.31)	265.27 (259.44 to 271.1)	<0.001	
	WP (cm)	86.68 (86.57 to 86.78)	100.89 (100.44 to 101.35)	<0.001	
		**No n = 17.991**	**Yes n = 745**		
		**Median (95%CI)**	**Median (95%CI)**	**p**	
Female	BMI (kg/m^2^)	24.34 (24.27 to 24.4)	31.06 (30.65 to 31.48)	<0.001	
	SBP (mm Hg)	113.26 (113.06 to 113.47)	133.64 (132.44 to 134.85)	<0.001	
	DBP (mm Hg)	69.66 (69.52 to 69.8)	82.34 (81.6 to 83.09)	<0.001	
	Cholesterol (g/L)	188.24 (187.73 to 188.76)	221.68 (218.87 to 224.5)	<0.001	
	HDL-cholesterol (g/L)	54.65 (54.52 to 54.78)	45.45 (44.98 to 45.93)	<0.001	
	Glucose (g/L)	82.73 (82.57 to 82.89)	96.47 (95.11 to 97.84)	<0.001	
	Triglycerides (g(L)	79.89 (79.36 to 80.41)	164.31 (157.96 to 170.65)	<0.001	
	WP (cm)	73.97 (73.84 to 74.09)	88.62 (87.71 to 89.53)	<0.001	
**Premorbid metabolic syndrome (IDF Criteria)**					
		**No n = 21,363**	**Yes n = 3,166**		
		**Median (95%CI)**	**Median (95%CI)**	**p**	
Male	BMI (kg/m^2^)	26.15 (26.1 to 26.2)	30.66 (30.51 to 30.82)	<0.001	
	SBP (mm Hg)	123.55 (123.36 to 123.75)	137.85 (137.31 to 138.39)	<0.001	
	DBP (mm Hg)	74.49 (74.35 to 74.63)	84 (83.64 to 84.37)	<0.001	
	Cholesterol (g/L)	190.19 (189.7 to 190.67)	221.63 (220.2 to 223.06)	<0.001	
	HDL-cholesterol (g/L)	50.88 (50.79 to 50.96)	43.31 (43.04 to 43.59)	<0.001	
	Glucose (g/L)	86.6 (86.42 to 86.78)	99.59 (98.82 to 100.36)	<0.001	
	Triglycerides (g(L)	104.05 (103.27 to 104.83)	238.35 (233.38 to 243.32)	<0.001	
	WP (cm)	86.33 (86.22 to 86.44)	99.95 (99.6 to 100.3)	<0.001	
		**No n = 17,626**	**Yes n = 1,110**		
		**Median (95%CI)**	**Median (95%CI)**	**p**	
Female	BMI (kg/m^2^)	24.21 (24.14 to 24.27)	30.94 (30.61 to 31.27)	<0.001	
	SBP (mm Hg)	112.88 (112.67 to 113.08)	133.1 (132.14 to 134.06)	<0.001	
	DBP (mm Hg)	69.42 (69.28 to 69.56)	81.96 (81.34 to 82.58)	<0.001	
	Cholesterol (g/L)	187.76 (187.25 to 188.28)	218.32 (216.08 to 220.55)	<0.001	
	HDL-cholesterol (g/L)	54.82 (54.69 to 54.96)	45.71 (45.33 to 46.09)	<0.001	
	Glucose (g/L)	82.59 (82.44 to 82.75)	94.07 (93.03 to 95.12)	<0.001	
	Triglycerides (g(L)	79.23 (78.72 to 79.75)	146.91 (142.01 to 151.82)	<0.001	
	WP (cm)	73.77 (73.65 to 73.9)	86.83 (86.2 to 87.47)	<0.001	

PMetS: premorbid metabolic syndrome; BMI: body mass index; SBP: systolic bold pressure; DBP: diastolic blood pressure; HDL: high density lipoproteins; WP: waist perimeter.

### Correlation and regression analysis

**Figure 2:**
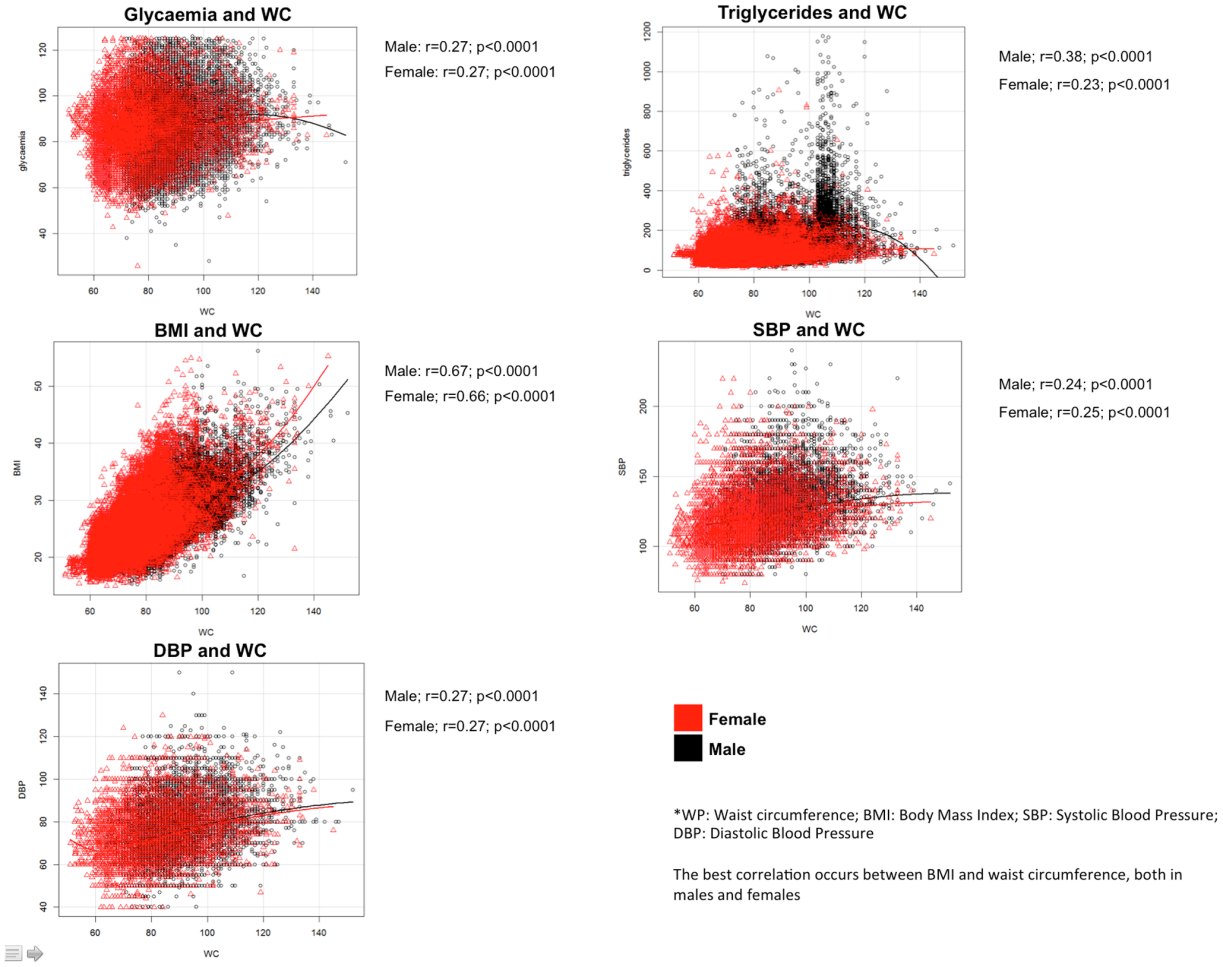
Relations of waist circumference with glycaemia, triglycerides, BMI and blood pressure by age categorized by gender.

Figure 2 shows the correlations between waist perimeter and the other four components of PMetS by gender. Significant correlations were found for all the parameters, both in male and females, but high correlational values were found only for BMI.

Logistic regression models were used to calculate adjusted ORs for PMetS with ATPIII and IDF criteria by age, gender, BMI and smoking status (Table 4). Results showed that overweight and obese participants had increased ORs compared to normal weight participants and current and former smokers increased ORs compared to non-smokers. Furthermore, lower PMetS risk was found in females and in the younger participants. The values of the ORs obtained using both ATPIII and IDF criteria were similar for all the risk factors analysed. The greatest difference was observed in the OR obtained for obese individuals, which is slightly higher using IDF criteria than using ATPIII ones. For both criteria, having an increased BMI was the factor increasing ORs more significantly. Regarding the smoking status, it is noteworthy that while using ATPIII criteria former smokers had the same OR as current smokers, whereas by using IDF criteria former smokers had a slightly higher OR than current smokers. Being a current smoker increased OR more using ATPIII criteria than using IDF ones. The Kappa agreement coefficient between IDF and ATPIII criteria was 0.845 (p<0.001).

**Table 4:** Adjusted ORs for PMetS determined following ATP III and IDF criteria by age, gender, BMI and smoking status.

ATP III (n = 43,265)		OR	95% CI
Age		1.067	1.06 to 1.07
Gender	Male (n = 24,529; 57.0%)	1.00	
	Female (n = 18,736; 43.0%)	0.37	0.34 to 0.41
BMI	Normal (n = 20,388; 47.1%)	1.00	
	Overweight (n = 15,343; 35.5%)	6.64	5.80 to 7.60
	Obesity (n = 6,994; 16.2%)	24.51	21.44 to 28.03
Smoking status	Non-smoker (n = 20,583; 47.6%)	1.00	
	Current smoker (n = 15,360; 35.5%)	1.66	1.53 to 1.80
	Former smoker (n = 7,322; 16.9%)	1.62	1.47 to 1.80
**IDF (n = 43,265)**		**OR**	**95% CI**
Age		1.068	1.06 to 1.07
Gender	Male (n = 24,529; 57.0%)	1.00	
	Female (n = 18,736; 43.0%)	0.42	0.39 to 0.45
BMI	Normal (n = 20,388; 47.1%)	1.00	
	Overweight (n = 15,343; 35.5%)	7.43	6.59 to 8.38
	Obesity (n = 6,994; 16.2%)	28.36	25.16 to 31.97
Smoking status	Non-smoker (n = 20,583; 47.6%)	1.00	
	Current smoker (n = 15,360; 35.5%)	1.48	1.37 to 1.59
	Former smoker (n = 7,322; 16.9%)	1.65	1.51 to 1.79

PMetS: premorbid metabolic syndrome; BMI: body mass index.

### Cardiovascular risk and cardiovascular risk factors

Regarding the cardiovascular risk factors, participants with PMetS showed significant higher values of BMI, waist circumference, systolic and diastolic blood pressure, total cholesterol, glucose and triglycerides serum, and significant lower HDL-cholesterol levels than participants without PMetS (Table 3).

Cardiovascular risk using the Framingham-REGICOR score was evaluated (Table 5), showing that 93.6% of subjects had a low risk profile (values of Framingham-REGICOR lower than 5%). Participants with PMetS showed a significantly higher cardiovascular risk than the ones without PMetS. Furthermore, the frequency of PMetS was higher in the participants with increased cardiovascular risk (Table 5). Age and gender adjusted OR for subjects with PMetS with ATP-III criteria was 12.33 (95% CI: 9.56 to 15.91) and 9.14 (95% CI: 7.07 to 11.82) with IDF criteria. Female subjects had lower cardiovascular risk profiles, with an OR = 0.13 (95% CI 0.08 to 0.19) for high or very high cardiovascular risk (p<0.001) with respect to males.

**Table 5:** Cardiovascular risk determined using Framingham-REGICOR equation.

		ATP-III criteria n (%)		IDF criteria n (%)	
Framingham-REGICOR	Global n (%)	PMetS	Without PMetS	PMetS	Without PMetS
<5% Low risk	40,500 (93.6)	2,123 (5.2)	38,377 (94.8)	3,032 (7.5)	37,468 (92.5)
5–10% Moderate risk	2,470 (5.7)	862 (34.9)	1,608 (65.1)	1,041 (42.1)	1,426 (57.9)
10–15% High risk	262 (0.6)	171 (65.3)	91 (34.7)	178 (67.9)	84 (32.1)
>15% Very high risk	33 (0.1)	24 (72.7)	9 (27.3)	25 (75.8)	8 (24.2)
**Total**	43,265 (56.7)	3,180 (7.4)	40,085 (92.6)	4,276 (29.9)	38,989 (90.1)

Comparison between ATP-III and IDF criteria.

Framingham-REGICOR >10% high CVD risk, 5–9.9% moderate CVD risk, <5% low CVD risk.

## Discussion

The results of the present cross-sectional study show a good agreement between IDF and ATPIII definitions, as well as a good association between the risk factors analysed and the presence of PMetS. BMI was found to be the main factor determining the presence of MetS for both ATPIII and IDF criteria. On the other hand, being female was found to be a protective factor. Regarding the relationship between PMetS and cardiovascular risk, higher levels of cardiovascular risk were found in participants with PMetS.

To the best of our knowledge this is the first epidemiological study on MetS prevalence in a large working Spanish population introducing the concept of “pre-morbid” to MetS and, thus, excluding participants suffering from type 2 diabetes and the ones with previous CVD events [31]. The sample size of the present study represents 9.45% of the total active working population in the Balearic Islands in 2011 [38]. Furthermore, the percentage of males and females in the sample is quite similar to what is observed in the whole active working population in the Balearic Islands (54.29% males and 45.71% females). Thus, the study sample could be considered as highly representative of the whole active working population. In fact, the sample analysed is the largest one considered in Spain in terms of determining not only MetS but also PMetS prevalence. This is one of the main strengths of the present study. On the other hand, as the main limitation, the cross-sectional design allowed us to describe only associations whereas no relationship over time could be obtained. Furthermore, participants highly concerned about their health, and thus probably healthier, along with those with a diagnosed disease, could represent the greater proportion of workers attending health examinations because these were not compulsory. This causes bias in the recruitment procedure as - in addition - it is not well-known whether the healthier workers or the ones with a diagnosed disease are the ones with the greatest interest in the checks. The self-reported data from participants could also be a limitation.

When comparing the results of the present study with published reports, it should be considered that most of the previous studies included older participants than the ones in the present study, which makes a comparison of the results difficult. A recent study performed in the USA reported an adjusted prevalence of MetS, using ATPIII criteria, of 20.6% in a working population including subjects older than 65 years [45]. The DECODE study, which included participants from nine European cohorts, showed a higher average prevalence of MetS: 32.2% in males and 28.5% in females. However, it should be considered that participants in this study were older, with an age range from 30 to 89 years [46]. More recent studies focusing on European populations, using ATPIII criteria, showed a similar prevalence of MetS, 25.9% in Norway [47], 28.8% in Turkey [48] and 24.7% in Luxembourg [20], all of which are higher than the one obtained in the present study. However, both the MESYAS [28] and ICARIA [30] studies revealed a lower prevalence of MetS than the ones indicated above and even than the one found in the present study. Differences could be related to the younger mean age of the participants included in both the MESYAS and ICARIA studies. Furthermore, the different adjustment method used in the studies hinders the interpretation of this low prevalence in relation to the one obtained in the present study and in the other studies indicated above.

Following WHO recommendations, the prevalence of PMetS was also determined in the present study. As could be expected, the exclusion of participants with diabetes mellitus or CVD induced a decrease in the prevalence of MetS. Two previous studies, DARIOS and HERMEX, determined the prevalence of PMetS in Spanish populations using IDF criteria [2], [27]. These studies showed, in small populations, a greater overall adjusted prevalence of PMetS than the one found in the present study. This lack of concordance in the results could be explained by the differences in age of the participants in the studies, as in the present study only the working population was considered and, thus, older individuals were not included in the sample. However, both the DARIOS and HERMEX studies included, on average, an older population (35–74 years in the DARIOS [27] and 25–79 in the HERMEX study [2]). In fact, the decrease in prevalence observed from MetS to PMetS in the present study was lower than the ones found in these previous studies [2], [27], which could also be related to the different age range considered, since the greatest differences between the prevalence of MetS and PMetS are observed in older age ranges.

A good agreement was found between ATPIII and IDF definitions for PMetS. However, the prevalence obtained using the IDF definition was slightly higher than the prevalence obtained using the ATPIII one. The greater prevalence found using the IDF definition could be mainly due to differences in the assigned cut-off points for abdominal obesity. A diagnostic of PMetS following the IDF definition can be made only when central obesity occurs, and the cut-off values used in this definition are lower than the ones used in the ATPIII definition. The fact that the IDF definition considers treatment for previously diagnosed hypertension and high levels of triglycerides as positive criteria - rather than only the presence of high blood pressure and high levels of triglycerides, as occurs in the ATPIII definition - could also account for the greater prevalence using IDF criteria.

Regarding the prevalence of PMetS categorized by gender, higher values were found in males than in females using both IDF and ATPIII criteria. A greater prevalence of PMetS in males than in females is a common finding [2], [27]. In fact, the DARIOS study reported an adjusted prevalence of PMetS of 26% in males and 24% in females [27]. These differences were confirmed in the HERMEX study which reported a prevalence of 23.5% in males and 18.5% in females [2]. However, the differences between genders observed in the present study (using IDF criteria) are much higher (26.15% in males and 9.19% in females) than in previous studies. In this sense, the HERMEX study showed that while the prevalence of PMetS in males was maintained throughout different age ranges between 45 and 79 years, in females prevalence increased dramatically in the older population, and was higher than in males in the oldest participants (65–79 years). In fact, the prevalence of MetS has been reported to be higher in older females than in men, in contrast to the effect of gender in younger adults [49]. Thus, the fact that in the present study this oldest population was not included could explain the significant difference found between genders in terms of PMetS prevalence.

Logistic regression analysis was performed to analyse the importance of variables such as age, gender, smoking status and BMI on the presence of PMetS in the Spanish population. Age, gender, smoking status and BMI were selected because they are not included in MetS criteria but all of them are of clinical relevance (age, smoking status, gender are cardiovascular risk factors and BMI is one of the parameters most used by clinicians in daily practice). Logistic regression analysis revealed lower risk of PMetS for females, non-obese subjects, non-smokers and younger participants. It is noteworthy that relationships found using ATPIII and IDF criteria were very similar. In this sense, BMI was found to be the main factor determining the presence of PMetS for both ATPIII and the IDF. This is in agreement with the results of a previous study developed in the USA focused on a working population, which showed a relationship between the presence of MetS and obesity with an OR = 25.94 [45]. On the other hand, being a female was found to play a protective role, which is in agreement with previous results [49]. In fact, most of the relationships found in the present study are coincident with the ones reported in previous studies using large populations. The smoking habit could be considered the main exception, because in the present study a greater prevalence of PMetS was found among smokers and former smokers whereas a previous study in a USA working population [45] reported that being a smoker or a former smoker was a protective factor.

Several epidemiological studies have confirmed the increased risk of CVD in the general population with MetS, independently of the diagnostic criteria used and how metabolic syndrome is associated with more cardiovascular events and all-cause mortality [8], [50]. The results of the present study show differences in cardiovascular risk depending on which PMetS criteria are used, with a significantly higher risk when the ATPIII definition is used. The mechanisms whereby MetS increases cardiovascular risk are not clear, but in the present study there is a strong association between the PMetS cluster of components and cardiovascular risk. However, studies performed in an older population [51] or in type 2 diabetic patients [52] found no association between MetS and cardiovascular risk. Conversely, in occupational populations, the association between MetS and increased cardiovascular morbidity and mortality has been confirmed [53].

The exclusion of participants suffering from type 2 diabetes and ones with previous CVD events enabled us to determine the prevalence of MetS in participants free of CVD and type 2 diabetes (PMetS), and the potential impact of primary prevention in the working population so that future preventive strategies can be focused on this population. Along these lines, it should be considered that there is some controversy as regards the usefulness of MetS over previous existing risk assessment tools [54]. Also, there is some controversy as to whether individual components of MetS are better predictors of cardiovascular disease than the whole group of components [55]. PMetS could improve the usefulness of MetS, allowing the early identification of people with a potential long-term risk. In fact, MetS has been reported to be linked to an increased risk of developing CVD [56] and type 2 diabetes [33]. This observation could even enhance the usefulness of PMetS in primary prevention.

Due to the fact that - as indicated above - the diagnosis of PMetS could enable the instauration of preventive strategies, the introduction of these strategies in the workplace could represent an interesting intervention strategy. In a systematic review, Groeneveld et al. (2010) analysed 31 RCTs of interventions in the workplace to reduce cardiovascular risk, concluding that such interventions were effective [57]. Through Healthy People 2010, the CDC recommended that at least 75% of workplaces should offer a comprehensive workplace health promotion program [58]. In this sense, there is evidence supporting the fact that the integration of programs promoting lifestyle modifications in the workplace enhances the effectiveness of these programs [59], with these effects going beyond the work place and exerting a positive influence on family environment [60], [61]. Furthermore, several studies have tested the efficiency of different interventions in the workplace in workers with MetS, showing, among other results, improvements in insulin resistance metabolic-related parameters [62] and adoption of healthier lifestyles [63].

## Conclusions

The presence of PMetS assessed using ATPIII and Framingham REGICOR is a useful approach for the early identification of apparently healthy individuals who have a significant risk of developing cardiovascular events and type 2 diabetes. This approach could be used to develop and validate a tool for the evaluation and primary prevention of type 2 diabetes and CVD in a working adult population in Spain and, thus, for educating the population on the control of modifiable risk factors as well as promoting healthy lifestyles to reduce comorbidities.

## Supporting Information

Table S1 **Crude and adjusted prevalence for Metabolic Syndrome in Balearic working population with ATPIII and IDF criteria.**
(DOC)
