# Supplementary material for: Prevalence of Premorbid Metabolic Syndrome in Spanish Adult Workers Using IDF and ATPIII Diagnostic Criteria: Relationship with Cardiovascular Risk Factors
Source: PLoS One. 2014 Feb 20;9(2):e89281. doi: 10.1371/journal.pone.0089281 (PMC3930690; doi:10.1371/journal.pone.0089281)
Supplement: Table S1 — Crude and adjusted prevalence for Metabolic Syndrome in Balearic working population with ATPIII and IDF criteria. (DOC) [file pone.0089281.s001.doc]

**Table S1. Crude and adjusted* prevalence for Metabolic Syndrome in Balearic working population with ATPIII and IDF criteria**

|  | ATP III | | **Global Crude prevalence** | | **95% CI** | | **Male crude prevalence** | | **95% CI** | | **Female crude prevalence** | | **95% CI** | | **Global adjusted prevalence** | | **95% CI** | | **Male adjusted prevalence** | | **95% CI** | | **Female adjusted prevalence** | **95% CI** | |
| --- | --- | --- | --- | --- | --- | --- | --- | --- | --- | --- | --- | --- | --- | --- | --- | --- | --- | --- | --- | --- | --- | --- | --- | --- | --- |
|  | **20-24** | | 43 (1,39) | | 1,18 to 1,6 | | 36 (2,04) | | 1,7 to 2,38 | | 7 (0,53) | | 0,33 to 0,72 | | 1,73% | | 1,47 to 2 | | 2,56% | | 2,14 to 2,98 | | 0,65% | 0,41 to 0,9 | |
|  | **25-29** | | 126 (2,3) | | 2,1 to 2,51 | | 97 (3,38) | | 3,04 to 3,72 | | 29 (1,12) | | 0,91 to 1,32 | | 3,81% | | 3,48 to 4,15 | | 5,15% | | 4,64 to 5,66 | | 2,04% | 1,67 to 2,42 | |
|  | **30-34** | | 291 (4,08) | | 3,85 to 4,32 | | 229 (5,82) | | 5,44 to 6,19 | | 62 (1,94) | | 1,7 to 2,19 | | 7,08% | | 6,68 to 7,48 | | 9,55% | | 8,95 to 10,15 | | 3,59% | 3,14 to 4,04 | |
|  | **35-39** | | 440 (6) | | 5,72 to 6,28 | | 355 (8,39) | | 7,97 to 8,82 | | 85 (2,74) | | 2,44 to 3,03 | | 10,44% | | 9,97 to 10,92 | | 14,08% | | 13,39 to 14,77 | | 4,94% | 4,42 to 5,46 | |
|  | **40-44** | | 583 (8,67) | | 8,33 to 9,02 | | 449 (11,76) | | 11,24 to 12,28 | | 134 (4,62) | | 4,23 to 5,01 | | 15,37% | | 14,79 to 15,96 | | 19,98% | | 19,14 to 20,83 | | 8,56% | 7,85 to 9,27 | |
|  | **45-49** | | 687 (11,64) | | 11,23 to 12,06 | | 540 (16,12) | | 15,49 to 16,76 | | 147 (5,76) | | 5,3 to 6,22 | | 20,05% | | 19,36 to 20,73 | | 27,09% | | 26,1 to 28,09 | | 10,18% | 9,39 to 10,98 | |
|  | **50-54** | | 654 (14,4) | | 13,87 to 14,92 | | 474 (17,92) | | 17,17 to 18,67 | | 180 (9,48) | | 8,81 to 10,16 | | 22,08% | | 21,32 to 22,84 | | 28,03% | | 26,93 to 29,12 | | 14,15% | 13,18 to 15,13 | |
|  | **55-59** | | 524 (18,49) | | 17,76 to 19,22 | | 386 (21,43) | | 20,47 to 22,4 | | 138 (13,36) | | 12,3 to 14,42 | | 20,84% | | 20,03 to 21,65 | | 27,05% | | 25,87 to 28,23 | | 12,71% | 11,7 to 13,72 | |
|  | **60-64** | | 251 (19,9) | | 18,78 to 21,03 | | 181 (21,91) | | 20,47 to 23,35 | | 70 (16,09) | | 14,33 to 17,85 | | 11,20% | | 10,54 to 11,87 | | 14,37% | | 13,38 to 15,36 | | 7,16% | 6,34 to 7,99 | |
|  |  | | **3599 (8,13)** | | **8 to 8,26** | | **2747 (10,88)** | | **10,69 to 11,08** | | **852 (4,47)** | | **4,32 to 4,62** | | **12,39%** | | **12,2 to 12,58** | | **21,39%** | | **21,07 to 21,7** | | **6,94%** | **6,71 to 7,17** | |
| IDF | | **Global Crude prevalence** | | **95% CI** | | **Male crude prevalence** | | **95% CI** | | **Female crude prevalence** | | **95% CI** | | **Global adjusted prevalence** | | **95% CI** | | **Male adjusted prevalence** | | **95% CI** | | **Female adjusted prevalence** | | **95% CI** |  |
| **20-24** | | 70 (2,26) | | 2 to 2,53 | | 52 (2,95) | | 2,55 to 3,35 | | 18 (1,35) | | 1,04 to 1,67 | | 2,82% | | 2,49 to 3,16 | | 3,70% | | 3,19 to 4,2 | | 1,68% | | 1,29 to 2,07 |  |
| **25-29** | | 177 (3,24) | | 3 to 3,48 | | 128 (4,46) | | 4,08 to 4,85 | | 49 (1,89) | | 1,62 to 2,15 | | 5,36% | | 4,97 to 5,75 | | 6,79% | | 6,21 to 7,37 | | 3,45% | | 2,97 to 3,94 |  |
| **30-34** | | 389 (5,46) | | 5,19 to 5,72 | | 295 (7,49) | | 7,07 to 7,91 | | 94 (2,94) | | 2,64 to 3,24 | | 9,46% | | 9 to 9,92 | | 12,30% | | 11,63 to 12,97 | | 5,44% | | 4,9 to 5,99 |  |
| **35-39** | | 580 (7,91) | | 7,59 to 8,22 | | 452 (10,69) | | 10,21 to 11,16 | | 128 (4,12) | | 3,76 to 4,48 | | 13,77% | | 13,24 to 14,3 | | 17,93% | | 17,16 to 18,69 | | 7,44% | | 6,81 to 8,07 |  |
| **40-44** | | 778 (11,57) | | 11,18 to 11,96 | | 580 (15,19) | | 14,61 to 15,77 | | 198 (6,82) | | 6,35 to 7,29 | | 20,52% | | 19,86 to 21,17 | | 25,81% | | 24,89 to 26,73 | | 12,65% | | 11,81 to 13,49 |  |
| **45-49** | | 890 (15,08) | | 14,62 to 15,55 | | 681 (20,33) | | 19,64 to 21,03 | | 209 (8,19) | | 7,65 to 8,74 | | 25,97% | | 25,22 to 26,72 | | 34,17% | | 33,1 to 35,23 | | 14,48% | | 13,55 to 15,4 |  |
| **50-54** | | 863 (19) | | 18,41 to 19,58 | | 618 (23,36) | | 22,54 to 24,19 | | 245 (12,91) | | 12,14 to 13,68 | | 29,13% | | 28,3 to 29,97 | | 36,54% | | 35,37 to 37,71 | | 19,27% | | 18,16 to 20,37 |  |
| **55-59** | | 689 (24,31) | | 23,51 to 25,12 | | 494 (27,43) | | 26,38 to 28,48 | | 195 (18,88) | | 17,66 to 20,09 | | 27,40% | | 26,51 to 28,29 | | 34,62% | | 33,36 to 35,88 | | 17,95% | | 16,79 to 19,12 |  |
| **60-64** | | 346 (27,44) | | 26,18 to 28,7 | | 245 (29,66) | | 28,07 to 31,25 | | 101 (23,22) | | 21,19 to 25,24 | | 15,44% | | 14,68 to 16,21 | | 19,46% | | 18,34 to 20,57 | | 10,34% | | 9,36 to 11,31 |  |
|  | | **4782 (10,8)** | | **10,65 to 10,94** | | **3545 (14,05)** | | **13,83 to 14,26** | | **1237 (6,49)** | | **6,32 to 6,67** | | **16,46%** | | **16,25 to 16,68** | | **28,42%** | | **28,07 to 28,76** | | **10,07%** | | **9,8 to 10,34** |  |

*Adjusted by Balearic population
